# First Report on CRISPR/Cas9-Based Genome Editing in the Destructive Invasive Pest *Tuta Absoluta* (Meyrick) (Lepidoptera: Gelechiidae)

**DOI:** 10.3389/fgene.2022.865622

**Published:** 2022-05-19

**Authors:** Shun-Xia Ji, Si-Yan Bi, Xiao-Di Wang, Qiang Wu, Yan-Hong Tang, Gui-Fen Zhang, Fang-Hao Wan, Zhi-Chuang Lü, Wan-Xue Liu

**Affiliations:** ^1^ State Key Laboratory for Biology of Plant Diseases and Insect Pests, Institute of Plant Protection, Chinese Academy of Agricultural Sciences, Beijing, China; ^2^ Agricultural Genome Institute at Shenzhen, Chinese Academy of Agricultural Sciences, Shenzhen, China

**Keywords:** CRISPR/Cas9, cinnabar, genome editing, Tuta absoluta, lepidoptera

## Abstract

The tomato leaf miner *Tuta absoluta* (Meyrick) is one of the world’s most destructive pests of tomato, and because of its severe economic impacts, as well as the development of pesticide resistance, the species has been intensively studied, especially in regard to the identification of targets for *T. absoluta* control. However, functional genomic studies of *T. absoluta* have been constrained by a lack of effective genetic tools. Therefore, the aim of the present study was to develop a CRISPR/Cas9 zygote microinjection protocol for generating heritable mutations in *T. absoluta*, using the ommochrome synthesis gene *cinnabar* as an easily evaluated target gene. The injection of fertilised eggs with Cas9 protein and four sgRNAs, which targeted *cinnabar* exon 3, resulted in a mutagenesis rate of 31.9% for eggs reaching adulthood, and *cinnabar* mutagenesis resulted in either red or mosaic eye colour phenotypes. As such, this study is the first to report a complete and detailed CRISPR/Cas9 workflow for the efficient genome editing of the globally important invasive pest *T. absoluta*. The application of this robust genome-editing tool to *T. absoluta* will greatly facilitate the discovery of suitable RNAi control targets and the subsequent development of novel control strategies.

## 1 Introduction

The tomato leaf miner *Tuta absoluta* (Meyrick) (Lepidoptera: Gelechiidae) is a destructive pest from Peru (Guillemaud et al., 2015), since the 1950s, has been recognised as one of the worst pests of tomato in South America (Garcia and Espul, 1982; Desneux et al., 2010). In 2006, *T. absoluta* was discovered in Spain, and since then, the species has been reported from over 90 countries and regions, including most of Europe, Africa, the Middle East, and Asia (Desneux et al., 2011; Guimapi et al., 2016; Biondi et al., 2018; Zhang et al., 2020; Li et al., 2021). In 2017, *T. absoluta* was detected in the Xinjiang Province of China (Zhang et al., 2020), which is one of the world’s main tomato-producing regions, and over the next 2 years, the pest had colonised ∼11,635.8 km (48.5%) of China’s roadways (Zhang et al., 2021) and was reported to cause severe tomato production losses, sometimes up to 100% (Urbaneja et al., 2012; Chen et al., 2021). Thus, *T. absoluta* represents a serious threat to global tomato production (Campos et al., 2017; Biondi et al., 2018; Mansour et al., 2018).

Because of the severe economic of *T. absoluta*, as well as the reported development of pesticide resistance (Roditakis et al., 2015, 2017; Siqueira et al., 2020; Langa et al., 2021), the species has been intensively studied, especially in regard to the identification of RNAi control targets. However, the limited efficacy duration of RNA interference (RNAi) (Rahmani and Bandani, 2021a, b) was constrained to use broadly it to do the functional genomic studies of *T. absoluta*. Therefore, more effective genetic approaches are urgently needed.

One potentially valuable strategy is the application of clustered regularly interspaced short palindromic repeat (CRISPR)/Cas9 genome-editing technology, which can be used to knock out genes, thereby overcoming the shortcomings of RNAi for functional gene studies. Previous applications of this technology to insect systems have usually used eye colour as a visual marker to facilitate mutant screening, and this has typically been achieved through mutagenesis of the *cinnabar* gene, which encodes an important enzyme (kynurenine hydroxylase) in the biosynthesis of ommochrome (Paton and Sullivan 1978; Green et al., 2012; Li et al., 2017; Xue et al., 2018). For example, *cinnabar*-targeting small guide RNA (sgRNA) molecules have been used to produce red eyes in *Nasonia vitripennis* (Li et al., 2017) and *Nilaparvata lugens* (Xue et al., 2018). However, even though the CRISPR/Cas9 system and *cinnabar* mutagenesis have been successfully used to generate and verify heritable mutations in other insects, respectively (Adrianos et al., 2018; Xue et al., 2018; Wang et al., 2020; Zhu et al., 2020; Zhan et al., 2021), their application to *T. absoluta* have yet not to be reported.

Accordingly, the aim of the present study was to develop a CRISPR/Cas9 zygote microinjection protocol for generating heritable mutations in *T. absoluta*, using the ommochrome synthesis gene *cinnabar* as an easily evaluated target gene. The results of the evaluation indicated that disruption of a *cinnabar* homolog in *T. absoluta* can induce both red and mosaic eye-colour phenotypes. Furthermore, this study is the first to present a complete and detailed CRISPR/Cas9 workflow for efficient genome editing in the economically important pest *T. absoluta*. The application of this robust genome-editing tool to *T. absoluta* will greatly facilitate the discovery of suitable RNAi control targets and the subsequent development of novel control strategies.

## 2 Materials and Methods

### 2.1 Insect Materials and Rearing

The *T. absoluta* population used for the present study was originally collected from Yuxi, Yunnan Province, China, in August 2018, and then reared on healthy tomato (*Lycopersicon esculentum* Mill, Maofen) plants in the laboratory (25–26 °C, 50–60% relative humidity), with a 14-h photoperiod.

### 2.2 RNA Isolation and cDNA Synthesis

Total RNA was extracted from *T. absoluta* using TRIzol reagent (Invitrogen, Carlsbad, CA, United States), according to the manufacturer’s instructions, and the quality of the resulting RNA was evaluated using both spectroscopy (Nano Photometer P330; Implen, Munich, Germany) and agarose gel electrophoresis. First-strand cDNA was then synthesised from 2 μg isolated RNA using the One-Step gDNA Removal and cDNA Synthesis Super Mix kit (TransGen, Beijing, China).

### 2.3 *Tacinnabar* Cloning and Sequence Analysis

A putative *cinnabar* gene of *T. absoluta* (i.e., *Tacinnabar*) was identified in *T. absoluta* transcriptome datasets (unpublished) by using blastp and tblastn analysis and a *cinnabar* homolog from *Bombyx mori* (NM_001112665.1) as queries with E-values ≤ 1 × ^−20^. Specific PCR primers (Table 1) were then designed, and PCR amplification of *Tacinnabar* was performed using FastPfu DNA Polymerase (TransGen, Beijing, China). The amplified fragments were purified using the AxyPrep DNA Gel Extraction Kit (Axygen, West Orange, NJ, United States) following the manufacturer’s instructions, cloned into the pEASY-Blunt Vector (TransGen, Beijing, China), and then sequenced.

**TABLE 1:** Summary of the *cinnabar* mutagenesis mediated by four sgRNAs in *Tuta absoluta*.

	sgRNAs + Cas9 Protein	RNase-free Water
Number of eggs injected	591	201
Number of eggs hatched	270	165
Hatching rate	45.7%	82.1%
Number of surviving adults	69	54
Number of mutated adults	22	0
Phenotypic mutation efficiency	31.9%	0.0%

The exon and intron boundaries of *Tacinnabar* were acquired from *T. absoluta* genomic DNA. Open reading frames (ORFs) were predicted using ORFfinder (http://www.ncbi.nlm.nih.gov/orffinder/). Meanwhile, both the molecular weight and isoelectric point of the corresponding protein were predicted using ExPASy (http://web.expasy.org/protparam/), and conserved functional domains within the protein sequence were identified using TMHMM 2.0 (http://www.cbs.dtu.dk/services/TMHMM-2.0/). Multiple amino acid sequence alignments were constructed using ClustalW, and a phylogenetic tree was constructed using the maximum likelihood method based on the Whelan and Goldman (WAG) model in MEGA7.0, with 1,000 bootstrap replicates.

### 2.4 sgRNA Design and *in Vitro* Synthesis

sgRNAs were designed using CHOPCHOP (http://chopchop.cbu.uib.no) based on the principle of GG (20 N) GG or CC (20 N) CC, where N is any nucleotide, and simultaneously evaluated for efficiency and potential off-target effects. Finally, four sgRNAs were selected in *Tacinnabar* exon 3 (Table 2).

**TABLE 2:** Primer sequences used in this study.

Primer	Primer Sequence (5´→3′)
Primers for Full-Length Gene Amplification	
F	CGGACTCGTGTTATTTA
R	GCACTTACCAATTAGAAA
Primers for screening mutant	
F	ATT​GAG​GAA​TCA​CCT​ACT​A
R	ACA​TCT​AAA​ACT​GTT​TAC​CA
Primers for synthesizing sgRNA	
sgRNA1-F	GAA​ATT​AAT​ACG​ACT​CAC​TAT​AGG​AGA​CAG​ACC​CCT​CTT​GTC​CGg​ttt​tag​agc​tag​aaa​tag​c
SgRNA2-F	GAA​ATT​AAT​ACG​ACT​CAC​TAT​AGG​AAT​TTG​GCG​CTG​TCT​GTG​CGg​ttt​tag​agc​tag​aaa​tag​c
SgRNA3-F	GAA​ATT​AAT​ACG​ACT​CAC​TAT​AGG​TGC​GAG​GGA​GAA​TGA​TAC​ACg​ttt​tag​agc​tag​aaa​tag​c
SgRNA4-F	GAA​ATT​AAT​ACG​ACT​CAC​TAT​AGG​ATG​ACA​TAC​CTT​ACG​ACG​CGg​ttt​tag​agc​tag​aaa​tag​c
sgRNA-R	AAA​AGC​ACC​GAC​TCG​GTG​CCA​CTT​TTT​CAA​GTT​GAT​AAC​GGA​CTA​GCC​TTA​TTT​TAA​CTT​gct​att​tct​agc​tct​aaa​ac

Synthetic sgRNA templates were generated using PCR and specific primers (Table 1), verified using agarose gel electrophoresis, and purified using the AxyPrep DNA Gel Extraction Kit. The templates were then transcribed *in vitro* using the MEGA script T7 High Yield Transcription Kit (Ambion, Austin, TX, United States), following the manufacturer’s instructions, and the synthesised sgRNAs were extracted using phenol/chloroform/isoamyl alcohol, diluted in RNase-free water, and stored at −80°C until further use.

### 2.5 Egg Microinjection

Eggs of mated females, which were collected within 3 h of oviposition, were quickly arranged on a microscope slide that was laminated with double-sided adhesive tape, and experimental eggs (*n* = 591) were injected with a mixture of sgRNAs (total 400 ng/μl, 100 ng/μl each sgRNA) and Cas9 protein (400 ng/μl; GenCrispr, Nanjing, China) using a microinjection system (Eppendorf, Hamburg, Germany), whereas control eggs (*n* = 201) were injected with RNase-free water. All eggs were then transferred to 90-mm Petri dishes, cultured under standard rearing conditions (as described above).

### 2.6 Phenotype and Genotype Evaluation

After hatching (4–5 days), larvae were transferred from the 90-mm egg dishes to 150-mm Petri dishes that contained fresh tomato leaves, and pupae were transferred to separate centrifuge tubes (1.5 ml) for sex differentiation. After eclosion, mutant female and male were paired in different cages (25 × 25 × 25 cm) to generate the F1 population.

The phenotypes of F0 adult specimens were evaluated by observing adult eye colour under a light microscope, and after mating and oviposition, genetic analysis was performed to evaluate *Tacinnabar* mutagenesis. More specifically, genomic DNA was extracted using the One Step Genotyping Kit (Vazyme, Nanjing, China) and used as a template for PCR amplification using gene-specific primers (Table 1). The amplified products were then sequenced directly, and if the peak diagram of the sequencing result showed that there were miscellaneous peaks near the target site, the PCR products were purified and cloned into the pEASY-Blunt Vector for monoclonal DNA sequencing. The same method was used to evaluate the mutant of F1 generation adults.

### 2.7 Homology Modelling of Mutated Protein

A homology model of the 18 bp-deletion mutant that generated by CRISPR/Cas9 tool in this study was generated using Swiss Model (https://swissmodel.expasy.org/interactive). Because the structure of Tacinnabar had not yet been solved empirically, a model of the wild-type Tacinnabar protein was first generated, and the result was used as a template to model the 18-bp deletion mutant.

## 3 Results

### 3.1 *Tacinnabar* Analysis

**FIGURE 1:**
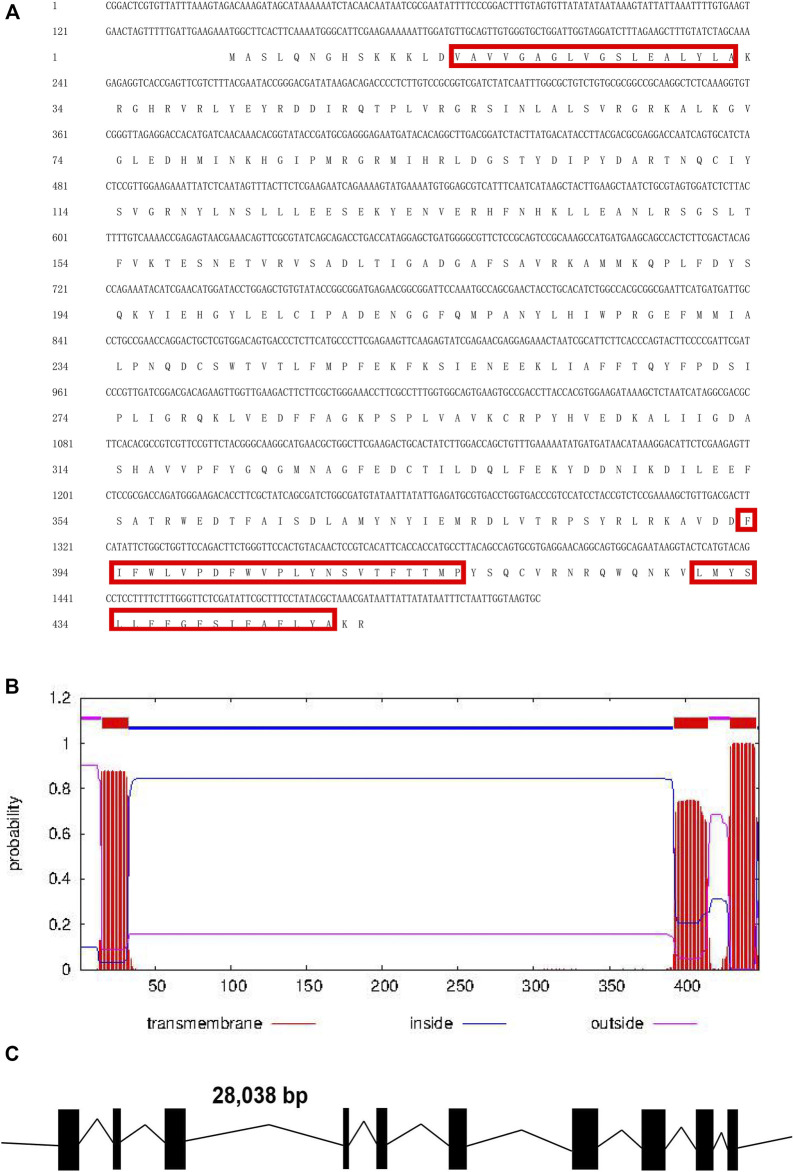
**(A)** Full-length cDNA sequence of *Tuta absoluta cinnabar* and its deduced amino acid sequence. Red boxes represented the three transmembrane domains. **(B)** Predicted conserved domains of the Tacinnabar protein. And three transmembrane domains were found in Tacinnabar. **(C)** Schematic of the exon/intron structures of the *Tacinnabar* gene. Solid blocks represented exons, and introns were indicated by full lines. The width of the solid blocks and the length of the black line were drawn in proportion to the actual length of the coding and non-coding regions, respectively.

The full-length *Tacinnabar* cDNA was 1,521 bp in length and contained a 142-bp 5ʹ untranslated region (UTR; positions 1–142), a 29-bp 3ʹ UTR (positions 1,493–1,521), and a 1350-bp ORF (positions 143–1,492), which encoded a 449-aa polypeptide with a predicted molecular mass of 51.8 kDa and isoelectric point of 7.16 (Figure 1A). Exon/intron structure analysis indicated that *Tacinnabar* contained 10 exons (Figure 1C), and the *Tacinnabar* amino acid sequence had three transmembrane domains (Figure 1B).

**FIGURE 2:**
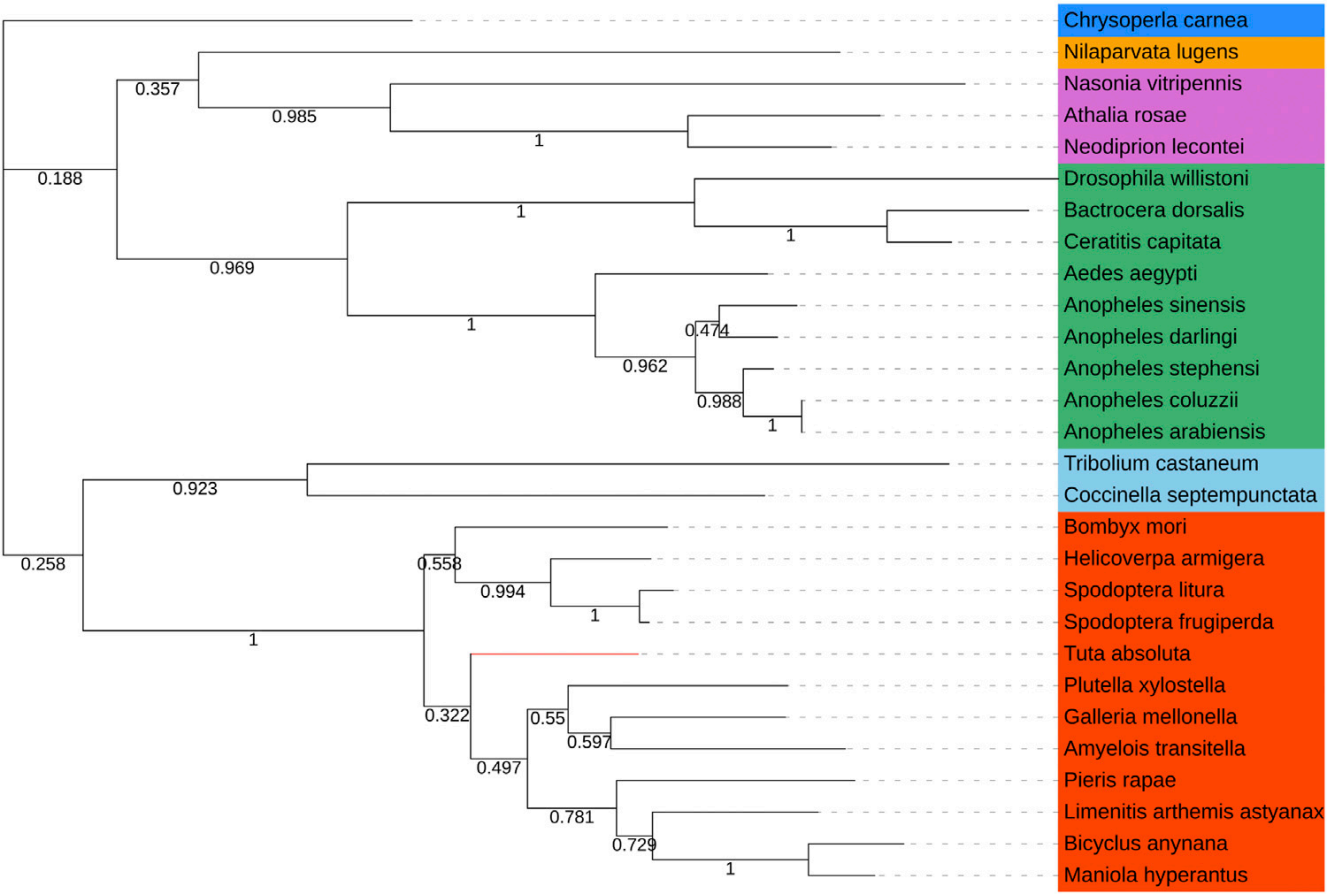
Phylogenetic tree based on the known amino acid sequences of *cinnabar* genes. The phylogenetic tree was constructed using the maximum likelihood method with 1,000 bootstrap replications in MEGA 7.0 Software. Values at the nodes are bootstrap values based on 1,000 replicates. Lepidoptera was depicted in red, Coleoptera in sky blue, Diptera in green, Hymenoptera in orchid, Hemiptera in orange, and Neuroptera in doderblue. *Helicoverpa armigera* (XP_021183543.1), *Spodoptera frugiperda* (XP_035436851.1), *Spodoptera litura* (XP_022820213.1), *Maniola hyperantus* (XP_034840116.1), *Limenitis arthemis astyanax* (QHN70696.1), *Amyelois transitella* (XP_013190001.1), *Bicyclus anynana* (XP_023953608.1), *Galleria mellonella* (XP_026757612.1), *Plutella xylostella* (XP_037974028.1), *Bombyx mori* (NP_001106135.1), *Pieris rapae* (XP_022118610.1), *Chrysoperla carnea* (XP_044742432.1), *Anopheles stephensi* (XP_035914333.1), *Anopheles arabiensis* (XP_040175243.1), *Anopheles darling* (ETN60831.1), *Anopheles coluzzii* (XP_040219701.1), *Anopheles sinensis* (KFB47145.1), *Aedes aegypti* (XP_001653516.2), *Ceratitis capitata* (XP_004522555.1), *Drosophila willistoni* (XP_015034226.1), *Bactrocera dorsalis* (XP_011207325.1), *Neodiprion lecontei* (XP_015524234.1), *Athalia rosae* (XP_025602652.1), *Nasonia vitripennis* (XP_001602258.1), *Coccinella septempunctata* (XP_044756441.1), *Nilaparvata lugens* (XP_039290505.1), *Tribolium castaneum* (NP_001034500.1).

We directly compared the *Tacinnabar* amino acid sequence to blastp sequences in NCBI, and it was found that the identity with known cinnabar sequences among 54 species exceeded 56%. In addition, the phylogenetic tree showed that cinnabar proteins of insects in each order clustered on a single branch. For example, phylogenetic analysis grouped the cinnabar proteins of *T. absoluta* and other lepidopterans (e.g., *Galleria mellonella, Amyelois transitella, Plutella xylostella*, and *Pieris rapae*) (Figure 2), which is consistent with the traditional taxonomy.

### 3.2 Evaluation of CRISPR/Cas9-Mediated Mutagenesis

**FIGURE 3:**
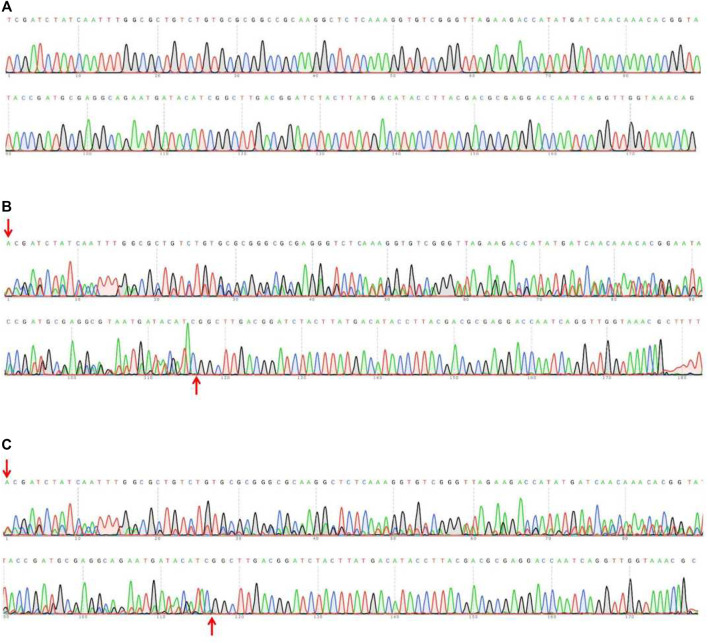
Sequencing assay on the PCR products in wild-type **(A)** and injected F0 **(B,C)**. The sequences of F0 mutations showed multiple peaks, indicating the occurrence of the mutations. The fragments between the red arrows represent the mutated sites.

**FIGURE 4:**
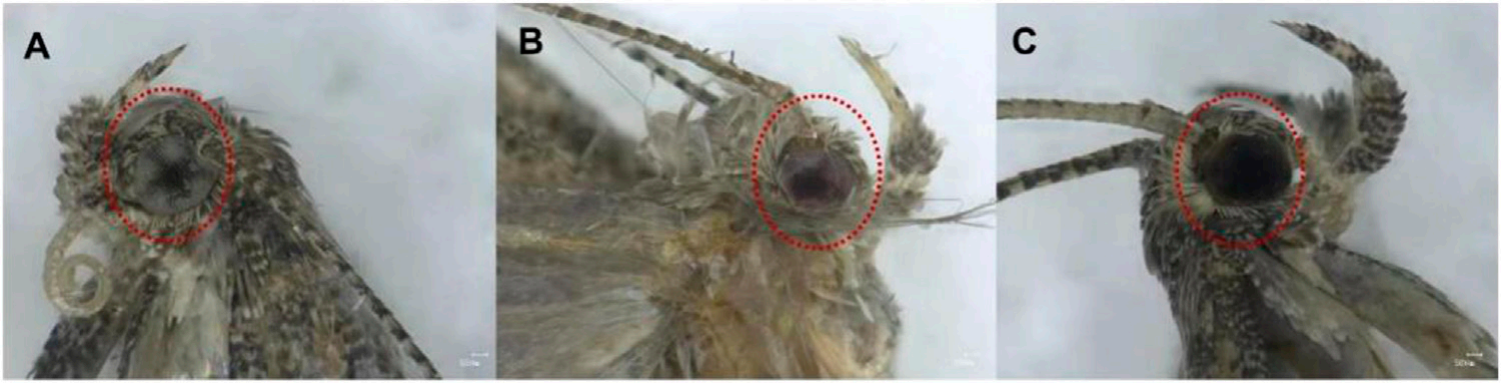
CRISPR/Cas9 induced mutations at the *cinnabar* gene in *Tuta absoluta* F1 individuals. **(A)** Wild-type eye colour in control *T. absoluta.* **(B)** *Cinnabar* mutant adults showed red eyes, and **(C)** mutant adults showed mosaic eye colour.

Table 1 showed that the 69 eggs (about 12%), from 591 eggs of microinjected sgRNAs + Cas9 proteins, developed successfully into adults. And there were 22 (31.9%) of the 69 adults were successful *Tacinnabar* mutagenesis (Table 1) and the mutation adults were verified (Figure 3), resulted in either red or mosaic eye colour in adults (Figure 4).

**FIGURE 5:**
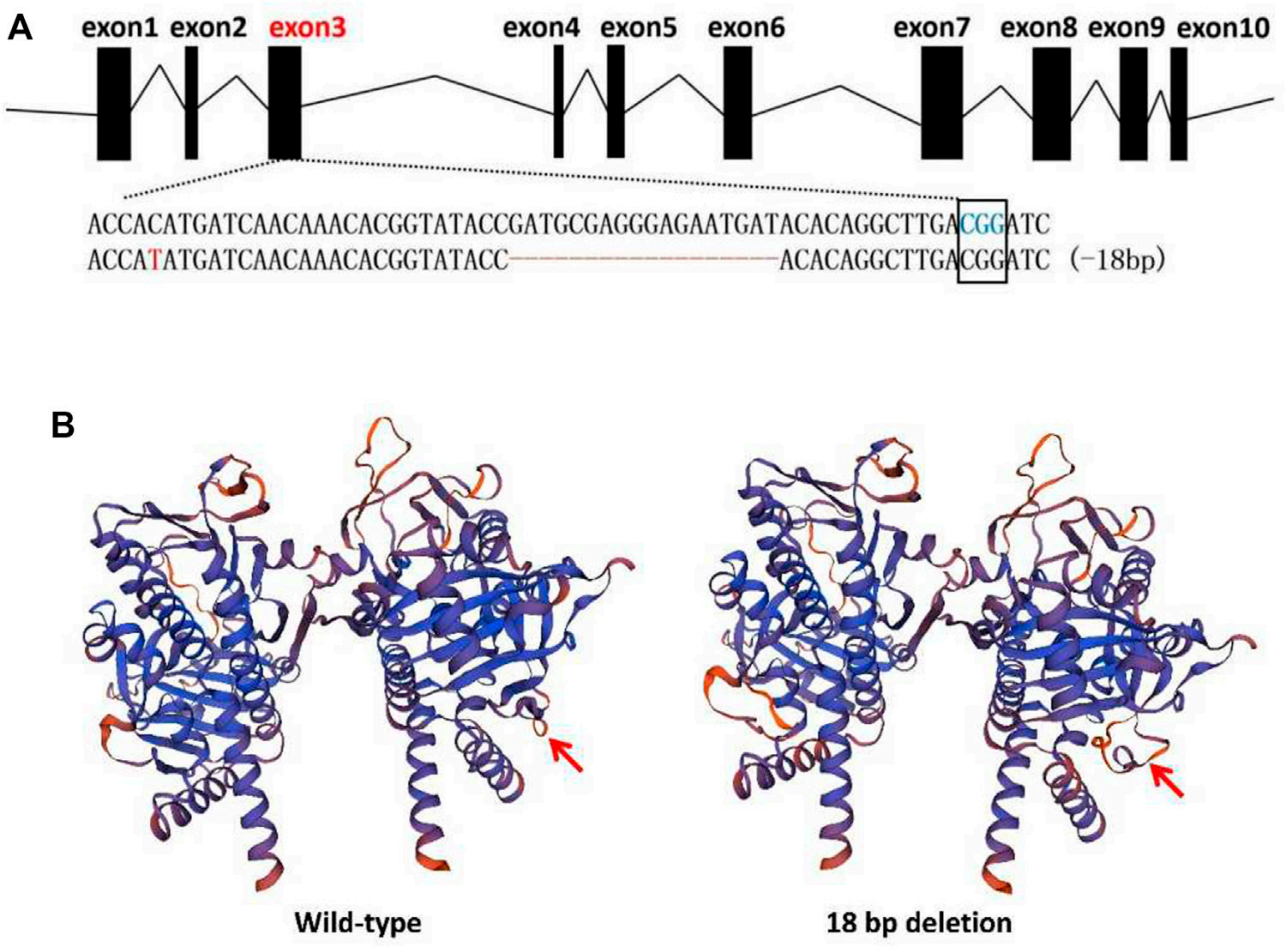
Mutant alleles and homology modelling of the 18 bp deletion in F1 generation. **(A)** The wild-type sequence was shown at the top with the target sites and the PAM (blue letters) was marked with black box. In mutant sequence, deletions were shown as red dashes and point mutation was shown as red letter. **(B)** Predicted 3D structure of the wild-type *Tuta absoluta* cinnabar protein (left) compared to the structure of the 18 bp deletion mutant (right). The deletion is predicted to cause the loop changes (red arrow).

**FIGURE 6:**
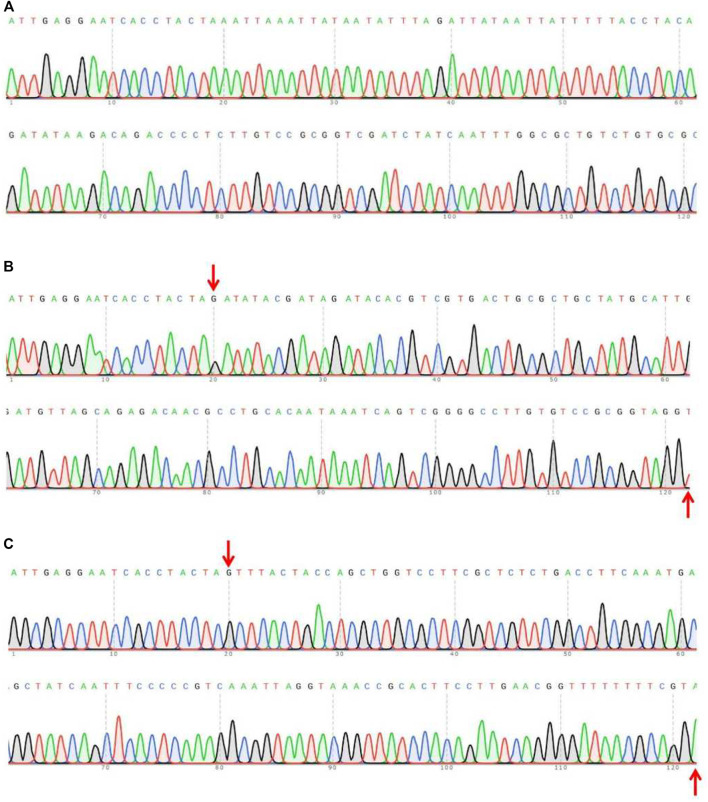
Part sequences of wild-type **(A)** and the garbled exon 3 of *Tuta absoluta cinnabar* in the F1 generation **(B,C)**. The fragments between the red arrows represent the garbled bases.

Furthermore, monoclonal sequencing of F1 mutants revealed that one of the adults harboured an 18-bp (GAT​GCG​AGG​GAG​AAT​GAT) deletion in *Tacinnabar* exon 3 (Figure 5A), which was predicted by homology modelling to cause loop changes in *Tacinnabar* (Figure 5B), and that other adults harboured altered bases throughout the entire third exon (196 bp) of *Tacinnabar* (Figure 6). Taken together, these data represented the success of the CRISPR/Cas9-mediated gene mutagenesis in the destructive worldwide pest *T. absoluta*.

## 4 Discussion

Recently, an increasing number of studies have focused on *T. absoluta* gene functions and the identification of target genes for RNAi-mediated control strategies (Camargo et al., 2016; Rahmani and Bandani, 2021a,b). Several studies have also been conducted in effort to improve RNAi efficiency, mainly by utilizing different dsRNA delivery methods (Majidiani et al., 2019; Bento et al., 2020). However, the application of RNAi to *T. absoluta* remains relative limitation. Therefore, the aim of the present study was to evaluate the efficacy of the CRISPR/Cas9 system.

As in other applications of CRISPR/Cas9 to insect systems, the present study focused on *cinnabar*, which regulates the synthesis of insect eye pigmentation, as a target gene, since its use would only require visual methods for mutagenesis screening (Paton and Sullivan, 1978). However, the *cinnabar* homolog in *T. absoluta* is unknown. As such, a full-length cDNA sequence of a *cinnabar* homolog (*Tacinnabar*) was identified and characterised in the present study. The Tacinnabar protein sequence contained three transmembrane domains and there were two transmembrane domains in *N. vitripennis* (NV14284) (Li et al., 2017) and *N. lugens* (KP881329) (Xue et al., 2018). However, little is known about the role of transmembrane domain number in ommochrome biosynthesis.

Interestingly, previous studies have demonstrated that the efficiency of CRISPR/Cas9-mediated mutagenesis can vary dramatically between species, target genes, and even sgRNAs that target the same gene. In general, mutagenesis rate is positively related to sgRNA and Cas9 concentrations (Bassett et al., 2013; Bi et al., 2016; Li et al., 2017), but efficiency can also be improved through the simultaneous use of multiple sgRNAs that target the same exon (Zhu et al., 2020), as in the present study. Indeed, the use of this strategy in the present study resulted in a *Tacinnabar* mutagenesis rate of 31.9% (22 of 69), which was higher than reported for the attempted mutagenesis of the *cinnabar* gene in *Nilaparvata lugens* (0%) (Xue et al., 2018).

Previous studies have also reported that *cinnabar* mutagenesis results in red-eye phenotypes (Li et al., 2017; Xue et al., 2018). However, the mutagenesis of *Tacinnabar* in the present study resulted in both red and mosaic phenotypes (Figure 6). It is possible that the injection of single *cinnabar*-targeting sgRNA molecules in *N. vitripennis* and *N. lugens*, which indued <30-bp insertions or deletions in *cinnabar*, only affected eye colour, whereas the simultaneous injection of four *cinnabar*-targeting sgRNA molecules resulted in larger-scale changes, including deletion of the whole third exon (196 bp), which could have induced different or more-pronounced effects on eye colour. On the other hand, the difference in mutant phenotypes could also be a result of differences in transmembrane domain number, which could mediate differences in the role of *cinnabar* in ommochrome synthesis in different insect species.

**FIGURE 7:**
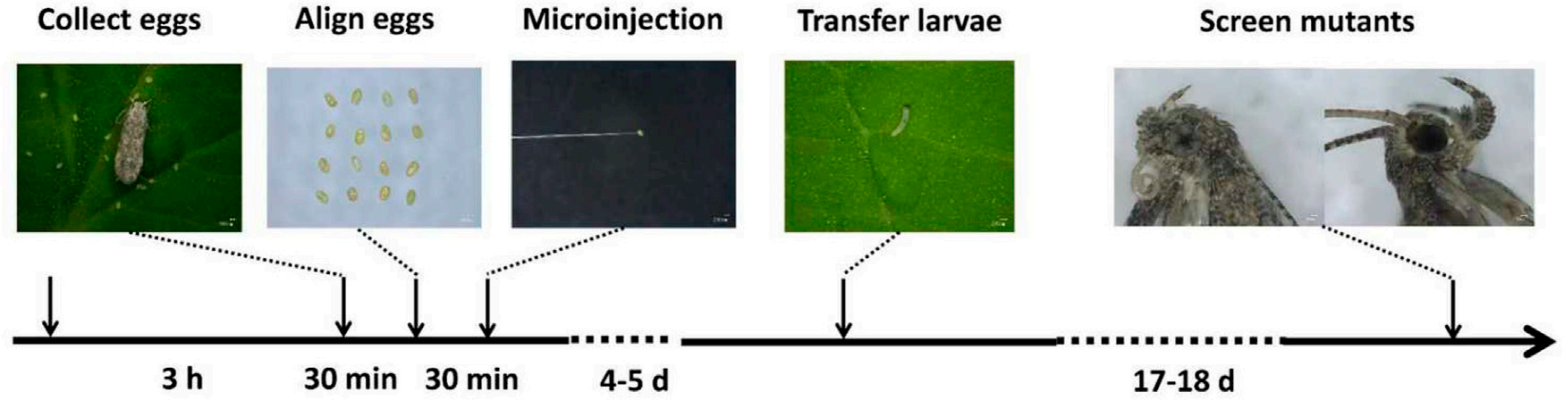
CRISPR/Cas9 workflow for the *Tuta absoluta*. Five stages: (1) adults laid eggs and collected all the eggs (within 3 h), (2) careful lined up the eggs on double-sided adhesive tape (with 30 min), (3) microinjection of embryos with a mixture of Cas9 protein and sgRNAs and placing back of injected embryos under rearing conditions (within 30 min), (4) careful transfer of newly hatched larvae on fresh tomato leaves (within 4–5 days) and (5) screening for the eye mutants after adult emergence (within 17–18 days).

The present study demonstrates, for the first time, that the CRISPR/Cas9 system can be used as an efficient means of gene editing in the globally important pest *T. absoluta* and describes a CRISPR/Cas9 workflow (Figure 7) that provides a more powerful strategy for reverse genetic studies, when compared to previous RNAi applications. The application of this robust genome-editing tool to *T. absoluta* will greatly facilitate the discovery of suitable RNAi control targets and the subsequent development of novel control strategies.

## Data Availability

The datasets presented in this study can be found in online repositories. The names of the repository/repositories and accession number(s) can be found below OM959367.
